# Comparative Pain Expression and Its Association to Intestinal Microbiota Through the MI-RAT© Osteoarthritis Model Induced in LOU/C/Jall and Sprague-Dawley Aged Rats

**DOI:** 10.3390/ijms26167698

**Published:** 2025-08-08

**Authors:** Marilyn Frézier, Colombe Otis, Emilie Labelle, Bertrand Lussier, Pierrette Gaudreau, Simon Authier, Marcio Carvalho Costa, Hélène Beaudry, Eric Troncy

**Affiliations:** 1 Groupe de Recherche en Pharmacologie Animale du Québec (GREPAQ), Université de Montréal, St.-Hyacinthe, QC J2S 2M2, Canada; marilyn.frezier@umontreal.ca (M.F.); colombe.otis@umontreal.ca (C.O.); sandemi15@hotmail.com (E.L.); bertrand.lussier@umontreal.ca (B.L.); simon.authier@crl.com (S.A.); marcio.costa@umontreal.ca (M.C.C.); helene.beaudry@umontreal.ca (H.B.); 2 Osteoarthritis Research Unit, University of Montreal Hospital Research Center (CRCHUM), Montréal, QC H2X 0A9, Canada; 3 Department of Medicine, Faculty of Medicine, Université de Montréal, Montréal, QC H3C 3J7, Canada; pierrette.gaudreau@umontreal.ca; 4 Charles River Laboratories, Laval, QC H7V 4B3, Canada

**Keywords:** musculoskeletal, aging, somatosensory sensitization, conditioned pain modulation, cognition, gut microbiota

## Abstract

To investigate the involvement of gut–brain axis in musculoskeletal chronic pain in the elderly, this preclinical study aimed to compare osteoarthritis (OA) pain expression, cognitive function and gut microbiota composition in two aging rat strains (11–15 months). A validated surgically induced OA model was used in Sprague-Dawley (SD; *n* = 12), as standard group, and in LOU/C/Jall rats (LOU; *n* = 8), a healthy aging model. The OA pain response was assessed longitudinally (60 days) through quantitative sensory testing (mechanical sensitization and endogenous inhibitory control functionality), spatial memory, and gut microbiota. At sacrifice, joint structural alterations and spinal neuropeptides concentrations were quantified. After OA induction, higher mechanical hypersensitivity in LOU than in SD was also associated with higher endogenous inhibitory control (*p* < 0.05). Expression of pro-/anti-nociceptive spinal neuropeptides, cognitive function and joint alterations were similar in both groups. Gut microbiota composition was different (*p* < 0.001) and different taxa were associated with each strain (e.g., *Akkermansia* spp. with LOU vs. *Lactobacillus* spp. with SD). This study suggests healthy aging to be associated with more efficient endogenous pain control and expression of specific intestinal bacteria. This research questions the implication of the intestinal microbiota in aging and chronic pain control.

## 1. Introduction

Osteoarthritis (OA) is the most prevalent degenerative joint disease in the aging population, especially among women, and represents a leading cause of disability and socio-economic burden worldwide [1]. Beyond its classical hallmarks—progressive cartilage degradation, bone remodeling with osteophytes formation and synovitis [2]—OA is now recognized as a chronic pain condition involving inflammatory and nociplastic processes [3]. Pain in OA evolves through peripheral and central sensitization, reflecting long-term neural plasticity [3,4,5,6,7]. Despite increasing knowledge of OA pathology, the mechanisms underlying chronic pain progression remain incompletely understood. Research often focuses on isolated components of the pain pathway, such as peripheral nociceptors, spinal circuits, or specific brain regions, without integrating how these systems interact. In this context, imbalances in endogenous pain modulation, including loss of inhibitory control or gain of facilitation, may underlie persistent pain states [6,8]. These mechanisms can be explored through quantitative sensory testing (QST), which is increasingly applied in humans and pets [8,9,10].

Validated preclinical OA models [11,12,13,14,15,16,17], such as the *M*ontreal *I*nduction of *R*at *A*rthritis *T*esting (MI-RAT©), reproduce chronic OA pain by combining cranial cruciate ligament transection and destabilization of the medial meniscus in young (2–4 months) female Sprague-Dawley (SD) rats [11,12]. This surgical model, refined by the addition of a calibrated exercise program, produces consistent histological joint degeneration, altered weight-bearing, a somatosensory phenotype and changes in spinal neuropeptide (NP) expression, mimicking human OA condition [13,17]. In particular, QST in the MI-RAT© model reveals (1) peripheral sensitization, with strong and persistent decrease in tactile paw withdrawal threshold (PWT) and (2) central sensitization, with a small number of stimuli necessary to trigger behavioral withdrawal on response to mechanical temporal summation and (3) modulation of pain, with an increase in conditioned pain modulation (CPM) illustrating the functionality of the endogenous pain inhibitory control [13,17]. These phenotypic changes in OA pain were consistent with changes in spinal pro- and anti-nociceptive NP, such as substance P (SP), calcitonin gene-related peptide (CGRP) somatostatin (SST) and enkephalins (Methionine- and Leucine-enkephalin, Met- and Leu-Enk, respectively) [13,17].

Aging introduces further complexity in pain modulation. With age, peripheral sensory neurons exhibit reduced responsiveness [18,19], but neuroplastic changes in central pathways—such as impaired descending inhibition—contribute to chronic pain maintenance [3,20]. This loss of inhibitory control or switch to facilitation may explain persistent pain in elderly individuals [21,22,23,24,25]. However, preclinical findings remain equivocal, with variability depending on pain models and test types [26,27]. Some studies reported that aged rodents have attenuated CPM [28], and others showed heightened hyperalgesia in older animals exposed to OA models, particularly females [29], potentially due to a disrupted descending inhibition from structures like the periaqueductal gray [30].

Interestingly, at the difference of inflammaging [31,32], the LOU/C/Jall (LOU) rat strain displays features of “healthy aging”, including preserved sensitivity and cognition into advanced age [33,34,35,36]. In contrast to standard aging observed in SD rats, old LOU rats (20–22 months) maintain mechanical sensitivity comparable to younger animals (4–6 months) [34]. This raises the hypothesis that healthy aging may involve a preserved or even enhanced facilitation/CPM. To test this, we compared LOU and SD rats’ pain responses following MI-RAT© OA induction, rather than comparing age groups within a single strain. This study was limited to the analysis of females to avoid sex-related variability.

The gut–brain axis has emerged as another potential regulator of chronic pain and healthy aging [37]. Recent studies report strong links between gut microbiota composition and pain modulation [38,39], via microbial metabolites, immune signaling, and the release of neuromodulators such as serotonin, gamma-aminobutyric acid (GABA), or opioids [40] [41]. Specific bacterial taxa have been associated with OA [32,42,43] and rheumatoid arthritis [43,44] in elderly patients, and age-related intestinal dysbiosis may promote systemic inflammation and chronic pain [32].

In this preliminary study, we aimed to characterize how pain expression and modulation evolve in two aging female rat strains—SD (standard aging) and LOU (healthy aging)—after OA induction using the validated MI-RAT© model. We hypothesized that pain expression (sensitization and CPM) would differ between LOU and SD rats. If this hypothesis of pain response was met, we further explored whether these differences aligned with spinal NP profile, cognitive function and gut microbiota composition. The first objective of this study was to compare the temporal evolution of OA pain expression using QST. The secondary objective was to investigate strain-specific cognitive function and gut microbiota composition after OA induction.

## 2. Results

All animals completed the study, and no complications were observed after the surgical procedures.

### 2.1. LOU Rats Exhibit Stronger and Longer-Lasting Mechanical Hypersensitivity After OA Induction

**Figure 1:**
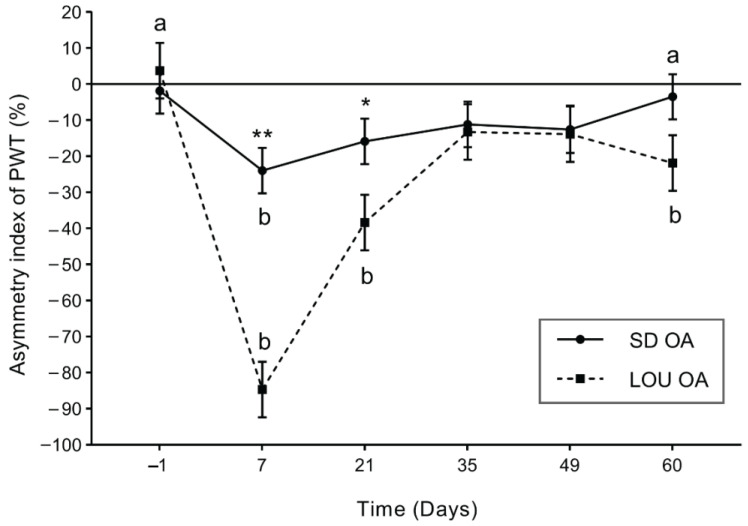
Temporal evolution of asymmetry index (%) of PWT (least squares mean ± standard error of the mean) in LOU (*n* = 8) and SD (*n* = 12) OA groups. Mixed model with Lognormal distribution. Fixed effects of group (*p* < 0.001), time (*p* < 0.001) and interaction of group by time (*p* < 0.001). Inter-group significant difference with adjusted *p*-value (*p*) (* *p* < 0.05; ** *p* < 0.01). Intra-group significant difference (a vs. b; *p* < 0.05).

After OA induction, the values of mechanical PWT right-to-left asymmetry index (Figure 1) decreased in both groups, more drastically at day (D) 7 (−84.63 ± 7.72%; *p* < 0.001) and D21 (−38.38 ± 7.72%; *p* < 0.05) for LOU than SD group (D7; −23.92 ± 6.30%; *p* < 0.01 and D21; −15.83 ± 7.72%). This means that both groups of rats have greater sensitivity in their ipsilateral (right) legs compared to their contralateral left (non-altered) legs. Moreover, after a respite, the allodynia in LOU resurfaced at D60 (*p* < 0.05) while allodynia observed in the SD group at D7 did not persist over time.

### 2.2. Enhanced Conditioned Pain Modulation in LOU OA Rats Suggests More Efficient Endogenous Inhibitory Control

**Figure 2:**
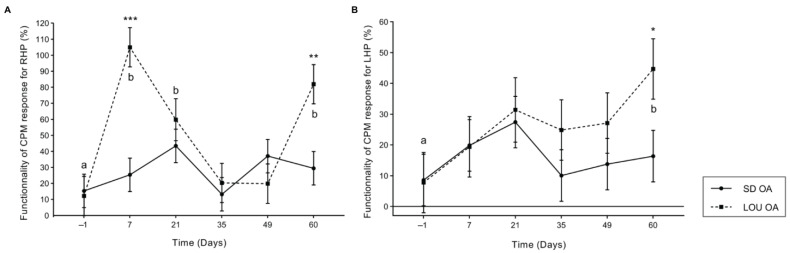
Percentage of change between tactile paw withdrawal threshold (PWT) post- vs. pre-conditioning stimulus (CS) for right-hind paw (RHP) (**A**) and left-hind paw (LHP) (**B**) by day (D) in LOU (*n* = 8) and SD (*n* = 11) OA groups (least squares mean ± standard error of the mean). Mixed model with Lognormal distribution. Fixed effects for RHP of group (*p* < 0.002), time (*p* < 0.001) and interaction of group by time (*p* < 0.001). Inter-group significant difference with adjusted *p*-value (*p*) (* *p* < 0.05; ** *p* < 0.01; *** *p* < 0.001). Intra-group significant difference (a vs. b; *p* < 0.01).

Figure 2 shows the CPM rate for SD and LOU groups for the right (OA-induced RHP, Figure 2A) and left (LHP, Figure 2B) hind paws. Statistical analysis indicated significant effect of group (*p* = 0.002), time (*p* < 0.001) and group × time interaction (*p* < 0.001) for RHP compared to no effect for LHP. For both groups, and both sides, the average CPM rate was positive at all timepoints. Regarding RHP (Figure 2A), LOU rats showed a significant increase, vs. baseline (BSL), in post-conditioning stimulus (CS) PWT after OA induction, at D7 (104.95 ± 12.26%; *p* < 0.001) and D21 (59.73 ± 13.10%; *p* < 0.01). Moreover, this effect decreased progressively in time to rise again at D60 (81.89 ± 12.26%; *p* < 0.001). The SD OA group showed a weaker modulation of pain, slightly stronger at D21 (43.39 ± 10.45%; *p* = 0.061). On the LHP side (Figure 2B), there was a significant within-time evolution only for the LOU OA group between BSL and D60 (*p* = 0.009). Also, both OA groups diverged from D21 up to D60 where the inter-group difference became significant (*p* = 0.030). 

At BSL, the percentage of responders to CPM (Table S1) tended to be higher in the SD OA group than the LOU OA group. After OA induction, the LOU OA rats had a trend to get more responders than SD OA, over time, on both sides, and particularly at D60 (100.00% vs. 90.91% for RHP and 100.00% vs. 63.64% for LHP, respectively). Whereas the rate of CPM responders was stable within-time (from the BSL) in the SD group, the increase was significant for the LOU group, at D21 and D60 for LHP (50.00% vs. 100.00%; *p* < 0.029).

### 2.3. Spinal Neuropeptide Changes in LOU OA Rats Reflect Global Sensitization but Do Not Distinguish Strain Differences

**Figure 3:**
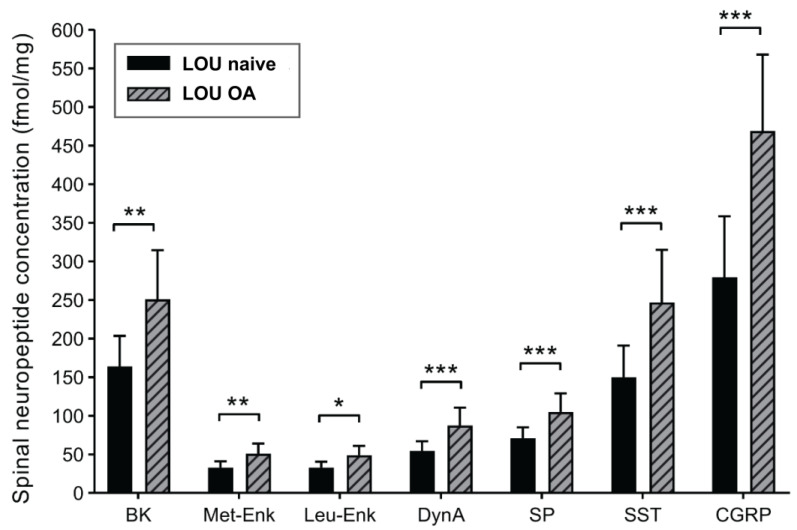
Spinal concentration (mean ± standard deviation) of BK, Met-Enk, Leu-Enk, DynA, SP, SST and CGRP at day 60 post-induction of OA in LOU OA (*n* = 8), and in LOU naive (*n* = 6) aging groups (17 months). Wilcoxon-Mann–Whitney test. Inter-group significant difference (* *p* < 0.05; ** *p* < 0.01; *** *p* < 0.001).

The targeted pain NP concentration was similar at D60 in both SD and LOU groups (Table S2). Figure 3 illustrates the D60 concentrations of the different spinal NP in aged LOU OA and LOU naive groups. Concentrations of excitatory NP, SP and CGRP and bradykinin (BK) were significantly increased in LOU rats after OA-induction (*p* ≤ 0.012) confirming the spinal sensitization. It was also true for inhibitory NP, such as Met-Enk, Leu-Enk, SST and dynorphin A (DynA) (*p* ≤ 0.015).

### 2.4. LOU and SD OA Rats Show Comparable Cognitive Search Strategies in Morris Water Maze Despite Locomotor Differences

Table 1 presents the parameters of the Morris water maze (MWM) test completed on LOU and SD groups (*n* = 8). Four rats from the SD OA group were excluded due to inconsistent target-crossing counts between manual and software-based analyses, combined with atypically short swim paths and deviating latency times, indicating outlier performance. Both groups of OA rats traveled greater distance and spent more time in quadrant 1 than in quadrant 3 in search of the platform (*p* < 0.001). The total distance traveled by LOU rats was greater than that of SD rats (2488.00 (189.80) cm vs. 2140.00 (257.10) cm; *p* = 0.007). At contrast, the speed, and the number of times that the rats enter the quadrant 1 were not different between OA rat groups.

### 2.5. Comparable OA-Induced Cartilage Damage Between Rat Strains

The histological modified Mankin’s score of LOU and SD stifle at sacrifice (D60), showed that the ipsilateral (right) stifle presented significantly more cartilaginous lesions than the contralateral stifle for both MI-RAT© groups (Total score; *p* < 0.029) (Table S3). The difference between ipsi- and contralateral stifle was due to more chondral lesions for LOU and SD (*p* < 0.034) and chondrocytes loss for SD only (*p* = 0.025) on the right side. On both sides, histological cartilaginous lesions were more elevated in the heavier SD rats (*p* < 0.015), but the ipsi- compared to contralateral lesion total score difference (≈2.60) was identical in both groups.

### 2.6. LOU and SD OA Rats Exhibit Distinct Gut Microbial Profiles with Divergent Diversity and Dominant Taxa

The gut microbiota data processing and cleaning yielded 2,702,331 sequences from 23 fecal samples for LOU OA rats and 4,379,271 sequences from 42 fecal samples for SD OA rats. One sample of SD OA rats at D60 was excluded from the analysis due to an insufficient number of reads. The mean of the Good’s coverage was 99.97% (min–max: 99.97–99.98%), demonstrating an adequate sampling effort at the chosen sub-sampling size. Since no significant temporal variation was observed within each group, all timepoints were pooled for the analysis and visualization of alpha and beta diversity indices.

**Figure 4:**
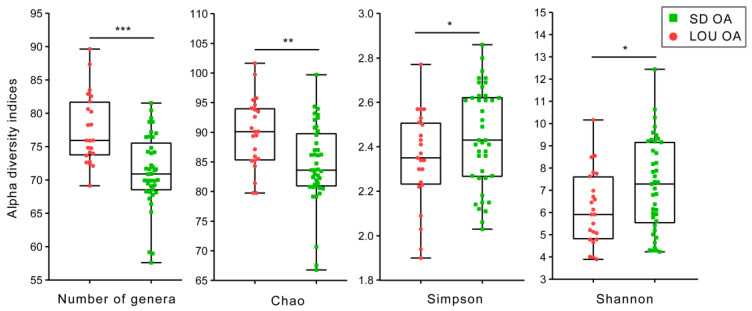
Comparison of gut microbiota alpha diversity between SD and LOU OA rat groups, showing species richness (number of genera and Chao’s index) and evenness (Shannon’s and Simpson’s indices) (mean ± min/max). Unilateral Student *t*-test. Significant inter-group statistical difference (* *p* < 0.05; ** *p* < 0.01; *** *p* < 0.001). The bacterial community membership (based on presence/absence; Figure 5A) and community structure (based on abundance; Figure 5B) differed significantly between the two OA groups (*p* < 0.001).

As shown in Figure 4, results of alpha diversity indices for bacterial communities revealed significant differences between SD (*n* = 12) and LOU (*n* = 8) OA rat groups. The LOU had higher richness than SD rats, as shown by the number of genera (*p* = 0.00002) or the Chao’s index (*p* = 0.002). However, the SD had higher evenness than LOU OA, as shown by the Simpson’s index (*p* = 0.02) or the Shannon’s index (*p* = 0.05). This finding indicates that LOU OA rats had more different bacteria, while SD OA had a more evenly distributed microbiota.

**Figure 5:**
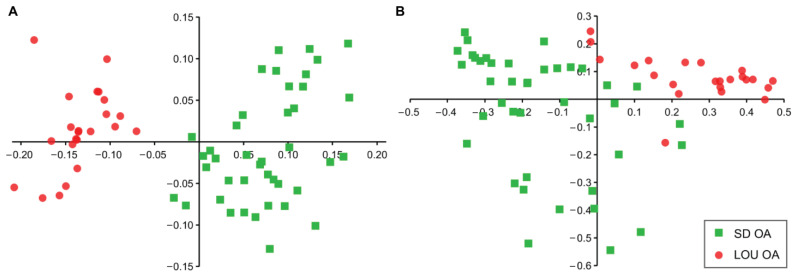
Principal Coordinate Analysis (PCoA) plots representing the dissimilarity between microbial communities of LOU OA and SD OA groups in feces samples for community membership addressed by the Jaccard index (**A**) and community structure addressed by the Yue and Clayton index (**B**). Analysis of molecular variance (AMOVA). Significant inter-group statistical difference (*p* < 0.001).

**Figure 6:**
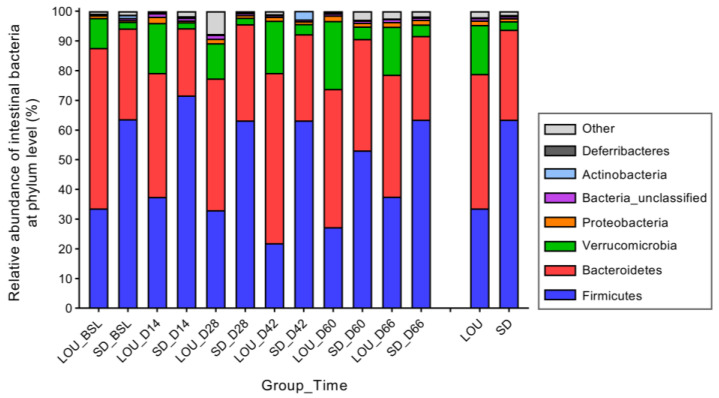
Temporal assessment of the relative abundances of bacterial communities found in feces of two OA rodent groups, SD and LOU, at the phylum level (median). Wilcoxon-Mann–Whitney test.

**Figure 7:**
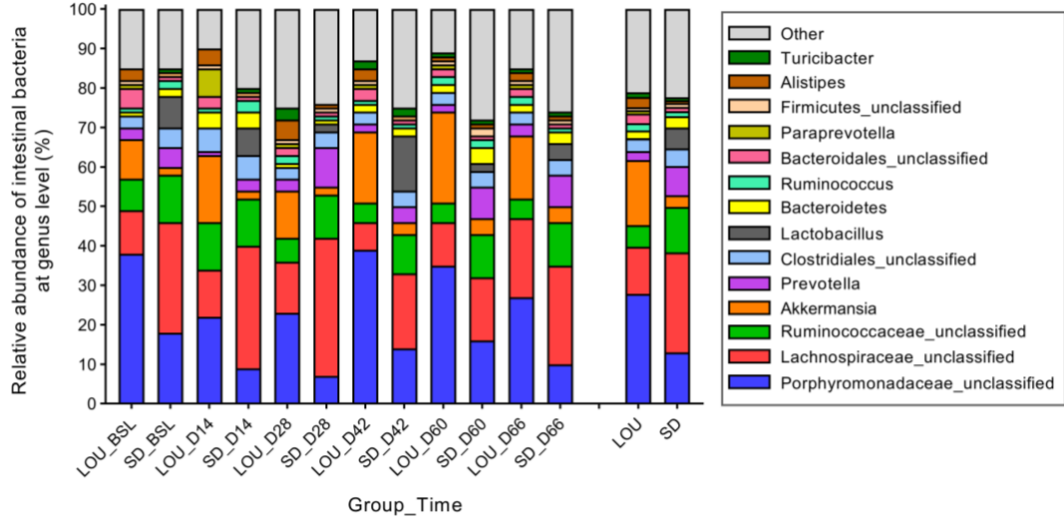
Temporal assessment of the relative abundances of bacterial communities found in feces of two OA rodent groups, SD and LOU, at the genus level (median). Wilcoxon-Mann–Whitney test.

The relative abundance of bacterial phylum (Figure 6) and genus (Figure 7) was different between LOU OA and SD OA rats. Firmicutes were greater in SD than in LOU rats (63.53% vs. 33.61%; *p* < 0.001), in contrast to the Bacteroidetes and Verrucomicrobia (30.27% vs. 45.31%, and 2.88% vs. 16.46%, respectively; *p* < 0.001). The Firmicutes/Bacteroidetes (F/B) ratio was higher in SD (2.10) than in LOU rats (0.67). At the genus level (Figure 6), there were huge global differences between LOU and SD OA groups: *Porphyromonadaceae* spp. (27.84% vs. 13.03%; *p* < 0.001), *Lachnospiraceae* spp. (11.95% vs. 25.31%; *p* = 0.002), *Ruminococcaceae* spp. (5.49% vs. 11.53%; *p* < 0.001), *Akkermansia* spp. (16.46% vs. 2.98%; *p* < 0.001), *Prevotella* spp. (2.30% vs. 7.41%; *p* = 0.003), *Clostridiales* spp. (3.17% vs. 4.39%; *p* = 0.001), *Lactobacillus* spp. (0.05% vs. 5.36%; *p* < 0.001) and *Alistipes* spp. (2.59% vs. 0.61%; *p* < 0.001).

**Figure 8:**
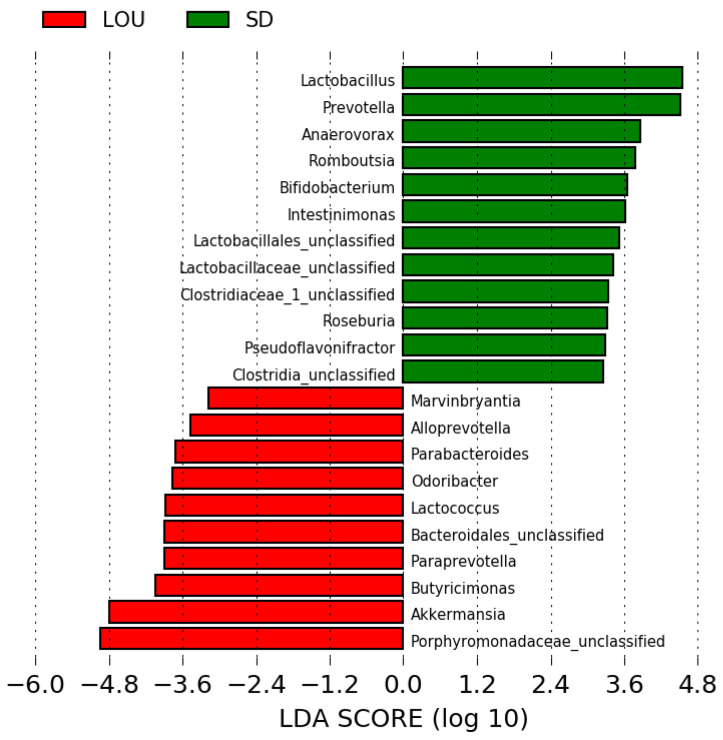
Linear effect size analysis indicating bacterial taxa significantly more abundant in fecal samples of either SD or LOU OA groups. Linear discriminant analysis (LDA) score > 3.0.

The linear effect size analysis confirmed that each rat strain was strongly associated with different bacteria (Figure 8). A member of the Porphyromonadaceae family and *Akkermansia* spp. were indicated as the strongest markers in the LOU OA, when *Lactobacillus* spp. and *Prevotella* spp. were for the SD OA group.

## 3. Discussion

The present study aimed to compare chronic pain phenotype—using behavioral (QST) and neurochemical (spinal NP) assessments—alongside cognitive function and gut microbiota profiles, between two aged female rat strains. These comparisons, within a validated OA model (MI-RAT©), provided potential new insights into how healthy aging might influence pain modulation and microbial composition through the gut–brain axis [37,38,39,40,41,44,45,46].

The MI-RAT© model, previously validated for mechanical hypersensitivity, temporal summation (RMTS), and CPM, effectively captured chronic OA pain and joint degeneration [11,12,13,17]. In this context, QST data revealed a pain centralized sensitivity profile peaking at D21, with larger CPM efficiency and a high percentage of CPM-positive responders. These enhancements declined over time, in line with the (quasi)normalization of weight-bearing and PWT at D49–56 in similar previous publications of the MI-RAT© model in young SD rats [11,12,13,17]. Spinal neuropeptidomic analysis at D56 confirmed the activation of both pro- (SP, CGRP, BK) and anti-nociceptive (SST) NP, alongside miR-181b upregulation, which is linked to reduced GABAergic inhibition and increased cartilage damage [17].

Across time, both strains of aged rats progressed from peripheral to centralized sensitization (Figure 1), consistent with existing data [13,17]. However, LOU rats displayed greater mechanical hypersensitivity at D7, D21, and D60 than SD rats. The latter showed limited responses beyond D7, reflecting human aging patterns in sensitivity [18,19]. Interestingly, LOU rats also exhibited stronger CPM engagement (around 82.20% vs. 40.22% in SD) bilaterally (Figure 2A,B). This dual-phase pattern—early hypersensitivity followed by sustained CPM—may reflect an adaptive pain modulation in LOU rats (“gain/loss” profile), while SD rats displayed a decline in sensitivity without compensatory inhibitory mechanisms (“loss/loss” profile). These findings echo reports in other OA models, such as chemical model in Lewis female rats [30], and support earlier results in young (2–4 months) SD rats showing impaired GABAergic signaling recognized in participating for the CPM response [17]. The PWT asymmetry index further reinforced this strain-specific sensitivity: aged LOU rats showed stronger asymmetry (−84.63 ± 7.72%) than young (−40.31 ± 6.48%) or old SD rats (−23.92 ± 6.30%) (Figure S1) [17]. Moreover, only LOU rats retained elevated CPM activation at D60 (Figure S2), indicating possible resilience with aging.

Despite these behavioral differences, spinal NP profiles at D60 did not significantly differ between strains (Table S2). Both showed upregulation of excitatory (BK, SP, CGRP) and inhibitory (Met-enk, Leu-enk, DynA, SST) neuropeptides (Figure 3), consistent with previous results in young SD rats [11,12,13,17]. This mismatch between phenotype and neurochemical profile suggests additional modulators, possibly cytokines, glial activity, or epigenetic mechanisms [47,48]. Moreover, the level of joint structural alterations was similar in both OA groups (Table S3), when normalized for the rat body weight, and the ipsi- to contralateral lesion total score ratio was identical in both groups. Spatial memory (MWM) scores were also similar across groups (Table 1), in contrast with earlier studies showing cognitive advantages in aged LOU rats [33,35,36]. This suggests that chronic OA pain may obscure cognitive differences in aging.

A key divergence emerged in gut microbiota profiles. Although both strains were fed distinct diets for extended periods, a veterinary nutritionist confirmed their equivalence in starch (43.00% vs. 44.20%) and fiber content, including neutral detergent fiber (14.92% vs. 14–15.00%), cellulose (3.55% vs. 3.20%), hemicellulose (10.43% vs. 9.80%), and lignin (0.94% vs. 1.00%) for LOU and SD, respectively. Since microbial structure is influenced by fiber and starch levels, nutrition is unlikely the sole driver of observed differences. Nonetheless, we acknowledge that long-term feeding histories and ingredient sources could have contributed. Still, the marked, consistent strain-specific differences in alpha (richness and evenness) (Figure 4) and beta (membership and structure) diversity (Figure 5), as well as taxonomic composition (Figure 6 and Figure 7), strongly suggest a physiological basis related to aging phenotype. Future studies should consider matched or standardized diets to limit nutritional confounders.

Alpha diversity analyses revealed higher richness in LOU OA rats but greater evenness in SD OA rats (Figure 4). In the literature, lower richness has been associated with dysbiosis and systemic inflammation in elderly subjects (“inflammaging”), while evenness alterations are variably linked to aging and chronic conditions like OA and neurodegeneration [31,32,49,50]. A mouse neuropathic model also reported decreased alpha diversity compared to controls [51]. In our dataset, the lower richness in SD rats may reflect a dysbiotic or pro-inflammatory gut environment. Conversely, the reduced evenness in LOU may relate to physiological aging rather than pathology [52,53]. These findings remain correlative and require further mechanistic clarification. Beta diversity analyses confirmed group-level differences in microbiota composition (Figure 5), with a higher F/B ratio in SD (>1)—a common dysbiosis marker—compared to LOU, which had a healthier ratio (close to 1) and more rare taxa (Figure 6, Figure 7 and Figure 8) [32,45,54,55].

At the genus level, SD OA rats were enriched in *Prevotella* spp., a genus associated with chronic inflammation and OA in humans [32,44,56]. *Prevotella* spp. can activate toll-like receptors and stimulate immune cells to produce pro-inflammatory mediators, reinforcing its role as a dysbiosis marker [40]. Interestingly, SD rats also showed high levels of *Lactobacillus* spp. and *Bifidobacterium* spp., typically regarded as beneficial [57]. These bacteria may support GABA production and reduce visceral pain via glutamate decarboxylase activity [58,59], and low *Lactobacillus* spp. has been associated with worse OA outcomes in SD rats [60]. However, a systematic review highlighted inconsistent findings regarding their efficacy in musculoskeletal conditions, especially in OA [61], so conclusions remain tentative. *Lachnospiraceae* spp., currently recognized as commensal bacterium, is associated with good health and appears to have a beneficial action on inflammation by inhibiting the expression of pro-inflammatory cytokines and nuclear factor kappa B [62,63].

In contrast, the LOU OA group was enriched in less-characterized genera like *Porphyromonadaceae* spp., *Akkermansia* spp. and *Butyricimonas* spp. *Porphyromonadaceae* spp. has been linked to healthier microbiota in visceral hypersensitivity models, suggesting a potential protective effect [64]. *Akkermansia* spp., particularly *Akkermansia muciniphila*, are known to support gut barrier integrity by reducing gut permeability and limiting pathogen translocation [65]. This protective effect has been associated with improved structural integrity of the intestinal epithelium, potentially through the preservation of tight junction proteins such as ZO-1 and occludin, which are key regulators of gut barrier function [66]. Studies have shown that the relative abundance of *A. muciniphila* declines with age in mice and macaques [67]. In contrast, its supplementation in aged animals promotes healthy aging, notably through anti-inflammatory effects and improved metabolic function [68]. Its presence in LOU rats may reflect gut barrier preservation, cognitive maintenance, and efficient pain modulation. This intriguing hypothesis warrants further investigation. *Butyricimonas* spp., also found in LOU rats, is a butyrate producer with anti-inflammatory and antidepressant potential, which may be relevant to chronic pain [69,70]. *Odoribacter* spp., another butyrate producer, has demonstrated analgesic properties and is under investigation as a probiotic, particularly in combination with *Akkermansia* spp. [71,72,73].

Altogether, these results suggest that healthy aging in LOU rats was associated with improved pain control and a distinct microbiota profile, despite equivalent severity of OA. This supports a multidimensional view of chronic OA pain shaped by both host aging phenotype and gut microbial composition.

Several limitations must be acknowledged. The small sample size, due to ethical considerations and limited access to aged LOU females, prevented the inclusion of young or sham-operated controls. The use of only female rats further restricts the generalizability of results. Notably, this work does not demonstrate a causal link between gut microbiota and pain modulation. Future work utilizing microbiota manipulation methods, such as cross-breed fecal microbiota transplantation, may provide further insights into the role of the intestinal microbiota in pain modulation.

## 4. Materials and Methods

### 4.1. Animals

The care and use of animals were subject to and approved by the Comité d’Éthique de l’Utilisation des Animaux de l’Université de Montréal (protocol_CEUA_Rech-1766) and the Comité Institutionnel de Protection des Animaux du Centre Hospitalier Universitaire de Montréal (CM14047PGr). The study was conducted in accordance with principles outlined in the current Guide to the Care and Use of Experimental Animals published by the Canadian Council on Animal Care and the Guide for the Care and Use of Laboratory Animals published by the US National Institutes of Health. This study is reported according to ARRIVE guidelines 2.0 [74]. This study was conducted on two rat strains: SD, obtained from Charles River Laboratories (formerly CitoxLAB), and LOU, which was not commercially available. They were generously provided by a collaborating laboratory (CRCHUM), which maintains an internal colony through the Quebec Network for Research on Aging (Montréal, QC, Canada). Due to this limited access, the sample size for this group was constrained. All rats were female housed in groups of two (SD) or two to three (LOU) rats, with water ad libitum and kept at a constant temperature of 22 °C in a 12-h light-dark cycle. The food for SD rats was Teklad Global Diet T2918.1^®^ (Envigo Inc., Indianapolis, IN, USA) and for LOU rats was R04-25^®^ kibbles (SAFE SAS, Augy, BFC, France). Rats (*n* = 20) were divided in two groups: (1) SD MI-RAT© (OA) group (*n* = 12; 426 (68) g; 11–15 months); and (2) LOU MI-RAT© (OA) group (*n* = 8; 192 (21) g; 11–15 months). Moreover, for the neuropeptidomic analysis, naive female LOU rats were included (*n* = 6; 192 (21) g; 17 months; no OA induction).

### 4.2. Montreal Induction of Rat Arthritis Testing (MI-RAT©) Model

On D0, intramuscular buprenorphine (1.0 mg/kg, Buprenorphine SR^®^, Chiron Compounding Pharmacy Inc., Guelph, ON, Canada) premedication preceded by 1-h general anesthesia with isoflurane gas (IsoFlo^®^, Abbott Animal Health, Montréal, QC, Canada) in O_2_ through an induction box first, followed by maintenance with face mask 2% isoflurane in O_2_ (1.0 L/min) mixture. After sterile surgical preparation, right stifle induction of OA was performed, as previously described and validated [11,12,13,17]. At the end of the surgery, a periarticular block of 0.25% bupivacaine (Marcaine^®^, McKesson Canada, St.-Laurent, QC, Canada) at the dose of 0.05–0.10 mL per stifle (<1 mg/kg) was performed to complete the opioid-based analgesia (buprenorphine). The MI-RAT© model includes a calibrated exercise protocol. All rats underwent a regular protocol of running (10 min each time) on a motor driven treadmill (IITC Life Science Inc., Woodland Hills, CA, USA) at a constant speed of 11 m/min, for three non-consecutive days a week for eight weeks. Calibrated exercise protocol in association with the surgical induction of OA was demonstrated to reduce the pain functional outcomes and structural OA joint lesions variability, mimicking human OA [13,17].

### 4.3. Experimental Design

Prior to evaluation, the rats were acclimatized to the evaluation environments at D–14, D–10, D–7, D–5 and D–3, according to a protocol previously validated [11,12,13,14,15,16,17]. Functional assessment timepoints were D–1 (BSL), D7, D21, D35, D49 and D60 post-OA induction. Both functional evaluation observers were women, blinded to the experimental design.

### 4.4. Mechanical Paw Withdrawal Threshold (PWT)

The secondary mechanical pain sensitivity involved using an electronic von Frey Esthesiometer^®^ with a propylene probe Rigid Tip^®^ of 0.7 mm^2^ surface, 28 G (IITC Life Sciences Inc., Woodland Hills, CA, USA). The determination of mechanical PWT of each hind paw was performed as described previously [11,12,13,14,15,16,17]. The peak force was recorded in grams, and a cut-off value was set at 100 g. Both hind paws were evaluated alternately, and triplicate measures were taken for each, with 60-second intervals between stimuli for each animal. The asymmetry index between the PWT of RHP and LHP was calculated using Equation (1) to assess mechanical hypersensitivity.

Asymmetry index of PWT (%) = [(PWT_RHP_ − PWT_LHP_)/((PWT_RHP_ + PWT_LHP_) × 0.5)] × 100 (1)

### 4.5. Conditioned Pain Modulation (CPM)

The CPM paradigm serves as a psychophysical experimental measure to evaluate the functionality of the pain descending endogenous inhibitory pathway [75,76,77]. It involves the application of a conditioning stimulus (CS) to decrease pain perception following an initial noxious stimulus [77]. In chronic pain condition, the dysfunction of the descending endogenous inhibitory control was shown, using CPM, in dogs with primary bone cancer and in rats with OA [17,78]. In rats, the CS was a curved Bulldog serrifine clamp clipped on left ear (50 mm in length, duration of 1 min), before performing a second PWT (post-CS) [17]. Functionality of CPM response was calculated with Equation (2).

Functionality of CPM response (%) = [(post-CS_PWT_ − pre-CS_PWT_)/pre-CS_PWT_] × 100 (2)

Functionality of the CPM response was determined too by looking at positive responders to CS. A rat was considered as a positive responder if its CPM response was higher than 0% (considered as no change).

### 4.6. Neuropeptidomic Analysis

Euthanasia was performed by rapid decapitation following isoflurane overdose (after the last functional evaluation day, D60) after which collection of the whole spinal cord was achieved by a saline flush technique [11,12,13,14,15,16,17]. The spinal cords from naive LOU rats were also collected to obtain comparative values from normal rats for each neuropeptide. Samples were snap frozen in liquid nitrogen, stored individually, and kept at −80 °C until neuropeptidomic analysis. All chemical solutions were purchased from Thermo Fisher Scientific (Toronto, ON, Canada). Each spinal cord was weighed accurately and homogenized using a Fisherbrand™ Bead Mill 24 Homogenizer (Thermo Fisher Scientific, Toronto, ON, Canada), following the addition of 0.25% trifluoroacetic acid solution at a ratio of 1:1 (*w*/*v*) in reinforced 1.5 mL homogenizer tubes containing 1 mm glass beads. The samples were homogenized with three bursts of 60 s at a speed of 5 m/s. An aliquot of 100 μL of the spinal cord homogenate was mixed with 100 μL of acetonitrile to precipitate high-molecular-weight proteins. The samples were vortexed and centrifuged for 10 min (12,000× *g*) and 100 μL of the supernatant was transferred into an injection vial, then was mixed with 100 μL of an internal standard solution containing labeled neuropeptides. After that, the neuropeptides concentrations were determined by the analytical method using the mass spectrometry coupled to high-performance liquid chromatography (HPLC-MS/MS) system as previously described [11,12,13,14,15,16,17].

### 4.7. Cognitive Evaluation

The MWM test is used to measure the neurocognitive function in particular spatial memory (University of Montreal Hospital Research Center (CRCHUM), Montréal, QC, Canada) [35,36]. It consists of placing distal markers on starting points around the perimeter of an open swimming arena with four quadrants. The animal must then swim from one of the markers to locate a submerged evacuation platform in quadrant 1. Repeated testing (three times ninety seconds) is used to assess spatial learning and reference memory is determined by preference for the area of the platform when the platform is absent. The MWM test was conducted as previously validated and published [35,36,79].

### 4.8. Structural Histological Joint Analysis

At sacrifice (D60), both stifle joints from all rats (*n* = 12 for SD and *n* = 8 for LOU) were collected and dissected. Then, the stifle joints were fixed in 10% formaldehyde solution (pH 7.4), decalcified, and embedded in paraffin for histological evaluation. Paraffined stifle sections, with thickness of 5 μm, were cut and hematoxylin and eosin and safranin-O/fast green stains were applied for histological evaluation. Articular lesions were graded on a scale using a table modified from Mankin’s score, based on direct microscopic observation by an independent blinded evaluator (see Table A1) [80,81]. The method was validated by a board-certified veterinary pathologist, consistent with our previous studies [13,17]. The table is composed of four criteria: chondral lesions scored from 0 (normal) to 10 (highest surface irregularities); proteoglycan loss evaluated with Safranin-O/fast green staining scored from 0 (no loss of staining) to 6 (loss of staining in all the articular cartilage by more than 50%); clusters formation scored from 0 (no cluster formation) to 3 (more than 8 clusters); and loss of chondrocytes scored on a 0 (normal) to 6 (diffuse loss of chondrocytes) scale. Total of alteration score was calculated by sum of the four criteria of Mankin’s score (0–25) for ipsilateral and contralateral stifle.

### 4.9. Gut Microbiota Analyses

Fecal samples were collected longitudinally from shared cages (two SD rats or two to three LOU rats per cage), prior to surgery and subsequently every two weeks after surgery. All samples were immediately stored at −80 °C until further processing. For each sample, 200 mg of fecal material was homogenized and filtered. DNA was extracted using a commercial kit (E.Z.N.A.^®^ Stool DNA Kit, Omega Bio-Tek Inc., Norcross, GA, USA), following the manufacturer’s instructions. The V4 region of the 16S rRNA gene was amplified using the primers: 515F (GTGCCAGCMGCCGCGGTAA) and 806R (GGACTACHVGGGTWTCTAAT). Amplicons were sequenced on the Illumina™ MiSeq platform (Illumina, San Diego, CA, USA) at the Genome Québec Innovation Centre (Montréal, QC, Canada).

Sequence data were analyzed using mothur software following a previously described Standard Operating Procedure [82,83,84]. High-quality reads were aligned with the SILVA reference database and clustered based on a 97% similarity. Chimeras were excluded with the vsearch tool in mothur and taxonomically classified using the Ribosomal Database Project (RDP).

Alpha diversity was assessed using the number of genera and the Chao index to estimate species richness, and the Shannon’s and Simpson’s indices to evaluate species evenness (diversity). Beta diversity was investigated by the Jaccard index to assess community membership (presence or absence of each taxon), and the Yue and Clayton index was used to compare community structure (presence and abundance of each taxon). The beta diversity analysis was visualized through principal coordinate analysis (PCoA).

Differentially abundant bacterial taxa at the genus level were identified using the Linear Discriminant Analysis Effect Size (LEfSe) with a logarithmic LDA score threshold of >3.0. This approach enabled comprehensive characterization of gut microbiota diversity and composition. For methodological reference, see [66,82,83,84].

### 4.10. Statistical Analysis

The sample size was determined by a statistical power calculation and by past similar experiments using the same stifle instability model of OA [11,12,13,17]. Statistical analyses for phenotype evaluation were achieved with a statistical software program (IBM^®^ SPSS^®^ Statistics Server version 29.0, New York, NY, USA), using an alpha value set at 0.05. Triplicate data for PWT and CPM were used as the average. Data of PWT and CPM were analyzed using general linear mixed models for repeated measures with the LogNormal distribution, correction for multiple comparisons and are presented as the estimate mean (least squares mean; LSM) with standard error of the mean (SEM) in figures. Treatment groups, time and their interaction (time × group) were considered as fixed effects. The difference between CPM positive responders has been tested with a Chi-square test. For NP, cognitive and histological analyses, data were presented as means with standard deviations and statistical differences were compared using the non-parametric Mann–Whitney-Wilcoxon bilateral test (GraphPad Prism^®^ Software version 8.0, Boston, MA, USA). For the inter-group comparison in gut microbiota of LOU and SD rats, a unilateral Student’s *t*-test was used for alpha diversity indices, and the analysis of molecular variance (AMOVA) was performed for beta diversity. All figures were made by GraphPad Prism^®^ Software.

## 5. Conclusions

This study compared pain responses and gut microbiota composition in two aged female rat strains after OA induction. The LOU strain, known for its association with healthy aging, exhibited stronger endogenous pain inhibition and a distinct microbial profile compared to standard SD rats. This is, to the authors’ knowledge, the first study to use QST and high-throughput sequencing to characterize these features in aged female LOU rats. The small sample size and the inclusion of only female rats restrict the generalizability of results. Although this work does not demonstrate a causal link between gut microbiota and pain modulation, the marked differences in bacterial profiles provide a rationale for further studies.

## Figures and Tables

**Table 1:** Parameters of test in MWM test (mean ± standard deviation) for LOU OA and SD OA rats. Wilcoxon-Mann–Whitney test (*p* < 0.05). Significant inter-group statistical difference (** *p* < 0.01). Significant intra-group statistical difference (a vs. b or c vs. d; *p* < 0.001).

MWM Test Parameters	LOU OA (*n* = 8)	SD OA (*n* = 8)
Time in quadrant 1 (s)	43.23 (9.79) ^a^	40.04 (8.17) ^a^
Time in quadrant 3 (s)	12.60 (4.51) ^b^	12.81 (6.96) ^b^
Distance in quadrant 1 (cm)	1118.00 (220.60) ^c^	898.80 (190.40) ^c^
Distance in quadrant 3 (cm)	385.00 (152.40) ^d^	333.20 (136.80) ^d^
Distance from the platform (cm)	54.62 (9.78)	51.11 (6.57)
Total distance swimming (cm)	2488.00 (189.80) **	2140.00 (257.10) **
Average speed in activity (cm/s)	34.68 (0.78)	34.64 (1.13)
Number of times the rats enter the area without platform (count)	2.75 (2.25)	2.88 (2.03)

## Data Availability

The datasets presented in this study are available in an online repository: https://data.mendeley.com/datasets/gn59wg845s/1, accessed on 15 January 2025.

## Supplementary Materials

The supporting information can be downloaded at: https://www.mdpi.com/article/10.3390/ijms26167698/s1.

## Abbreviations

The following abbreviations are used in this manuscript:

GREPAQ	Groupe de recherche en pharmacologie animale du Québec
CRCHUM	University of Montreal hospital research center
OA	Osteoarthritis
SD	Sprague-Dawley
LOU	LOU/C/Jall
QST	Quantitative sensory testing
MI-RAT©	Montreal induction of rat arthritis testing
NP	Neuropeptides
PWT	Paw withdrawal threshold
CPM	Conditioned pain modulation
SP	Substance P
CGRP	Calcitonin gene-related peptide
SST	Somatostatin
Met-Enk	Methionine-enkephalin
Leu-Enk	Leucine-enkephalin
GABA	Gamma-aminobutyric acid
D	Day
RHP	Right hind paw
LHP	Left hind paw
BSL	Baseline
CS	Conditioning stimulus
BK	Bradykinin
DynA	Dynorphin A
MWM	Morris water maze
PCoA	Principal coordinate analysis
AMOVA	Analysis of molecular variance
F/B	Firmicutes/Bacteroidetes
LDA	Linear discriminant analysis
LSM	Least squares mean
SEM	Standard error of the mean

## Appendix A

**Table A1:** Modified Mankin’s score table for histological analysis of joint alterations (adapted from [80,81]).

I. Structural changes (0–10)	
Normal 0	0
Surface irregularities (Undulating articular surface but no fibrillation)	1
Minimal mild superficial fibrillation (less than 10% of articular cartilage thickness) < 50%	2
Minimal mild superficial fibrillation (less than 10% of articular cartilage thickness) > 50%	3
Fibrillation/clefts/fissure/loss of articular cartilage involving superficial 1/3 of articular cartilage < 50%	4
Fibrillation/clefts/fissure/loss of articular cartilage involving superficial 1/3 of articular cartilage > 50%	5
Fibrillation/clefts/fissure/loss of articular cartilage involving superficial 1/3 to 2/3 of articular cartilage < 50%	6
Fibrillation/clefts/fissure/loss of articular cartilage involving superficial 1/3 to 2/3 of articular cartilage > 50%	7
Fibrillation/clefts/fissure/loss of articular cartilage involving superficial > 2/3 of articular cartilage < 50%	8
Fibrillation/clefts/fissure/loss of articular cartilage involving superficial > 2/3 of articular cartilage > 50%	9
Fibrillation/clefts/fissure/loss of articular cartilage to subchondral bone	10
II. Safranin O staning (0–6)	
Normal	0
Loss of staining in superficial zone of articular cartilage involving <50%	1
Loss of staining in superficial zone of articular cartilage involving ≥50%	2
Loss of staining in upper 2/3 of articular cartilage involving <50%	3
Loss of staining in upper 2/3 of articular cartilage involving ≥50%	4
Loss of staining in all the articular cartilage involving <50%	5
Loss of staining in all the articular cartilage involving >50%	6
III. Cluster formation (0–3)	
None	0
<4 clusters	1
≥4 but <8 clusters	2
≥8 clusters	3
IV. Loss of chondrocytes (0–6)	
Normal	0
Focal chondrocyte loss	1
Loss of chondrocytes in superficial zone < 50%	2
Loss of chondrocytes in superficial zone > 50%	3
Loss of chondrocytes in mid-zone < 50%	4
Loss of chondrocytes in mid-zone > 50%	5
Diffuse loss of chondrocytes	6
