# Supplementary material for: Comparative Pain Expression and Its Association to Intestinal Microbiota Through the MI-RAT© Osteoarthritis Model Induced in LOU/C/Jall and Sprague-Dawley Aged Rats
Source: Int J Mol Sci. 2025 Aug 8;26(16):7698. doi: 10.3390/ijms26167698 (PMC12386630; doi:10.3390/ijms26167698)
Supplement: Supplementary file 1 [file ijms-26-07698-s001.zip › ijms-3706778-supplementary.pdf]

Table S1. Percentage of positive responders to CPM (mean) for LOU OA and SD OA rats in RHP and LHP. Chi-square test ($p < 0.05$). Intra-group significant difference (a vs. b; $p < 0.029$).

Timepoints (days)	-1 (BSL)	7	21	35	49	60
RHP						
SD OA ($n = 11$)	90.91	81.82	100.00	90.91	72.73	90.91
LOU OA ($n = 8$; $n = 7$ at D21)	62.50	100.00	100.00	62.50	75.00	100.00
LHP						
SD OA ($n = 11$)	81.82	81.82	90.91	72.73	54.55	72.73
LOU OA ($n = 8$; $n = 7$ at D21)	**50.00** [a]	50.00	**100.00** [b]	62.50	75.00	**100.00** [b]

Table S2. Spinal concentrations, mean (standard deviation), of bradykinin (BK), methionine enkephalin (Met-Enk), leucine enkephalin (Leu-Enk), dynorphin A (DynA), substance P (SP), somatostatin (SST) and calcitonin gene related-peptide (CGRP) at day 60 post-induction of OA in LOU and SD old groups (11–15 months). Wilcoxon-Mann-Whitney test ($p < 0.05$).

Concentration (fmol/mg)	SD OA ($n = 12$)	LOU OA ($n = 8$)
BK	287.050 (73.702)	251.304 (63.264)
Met-Enk	58.168 (14.225)	51.131 (13.171)
Leu-Enk	57.157 (13.908)	49.270 (12.215)
DynA	101.595 (26.506)	87.673 (23.263)
SP	119.634 (28.217)	105.133 (24.153)
SST	284.721 (80.643)	247.145 (68.048)
CGRP	507.883 (133.803)	469.059 (99.076)

Table S3. Histological modified Mankin's score, mean (standard deviation), for LOU OA and SD OA rats in right (RHP) and left (LHP) stifles at sacrifice (D60). Wilcoxon-Mann-Whitney test ($p < 0.05$).

Modified Mankin's score	LOU OA (n = 8)			SD OA (n = 12)			LOU *vs.* SD	
Mean (SD)	RHP	LHP	*p*-value	RHP	LHP	*p*-value	*p*-value RHP	*p*-value LHP
I. Chondral lesions (0–10)	1.50 (1.07)	0.00 (0.00)	**< 0.001**	2.33 (2.39)	1.75 (2.96)	**0.034**	0.295	**0.015**
II. Proteoglycan loss (0–6)	0.50 (1.07)	0.00 (0.00)	0.467	1.08 (1.00)	0.58 (0.79)	0.242	0.145	0.087
III. Clusters formation (0–3)	0.50 (0.53)	0.00 (0.00)	0.077	0.42 (0.90)	0.33 (0.89)	0.932	0.356	0.495
IV. Chondrocytes loss (0–6)	0.13 (0.00)	0.00 (0.00)	1.000	2.58 (1.31)	1.17 (1.59)	**0.025**	**< 0.001**	0.087
Total score (0–25)	2.63 (1.60)	0.00 (0.00)	**< 0.001**	6.42 (3.90)	3.83 (5.75)	**0.029**	**0.008**	**0.015**

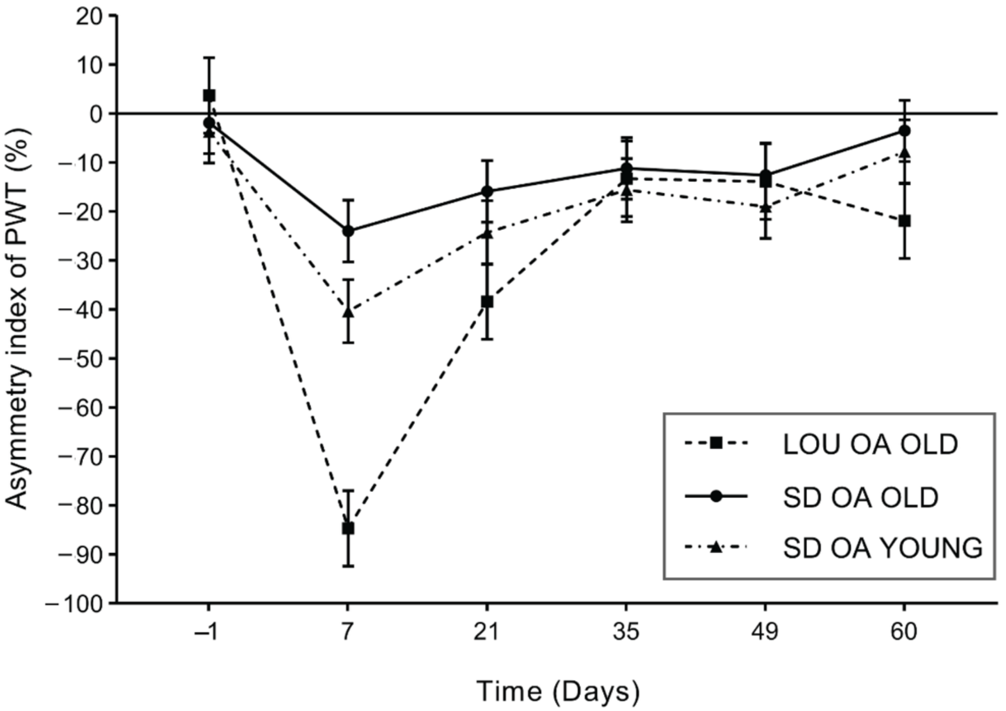

Figure S1. Temporal evolution of asymmetry index (%) of paw withdrawal threshold (PWT) in LOU old (n = 8; 11–15 months) and SD old (n = 12; 11–15 months) OA groups in comparison with SD young (n = 12; 2–4 months) OA group. Descriptive data (mean (standard deviation)).

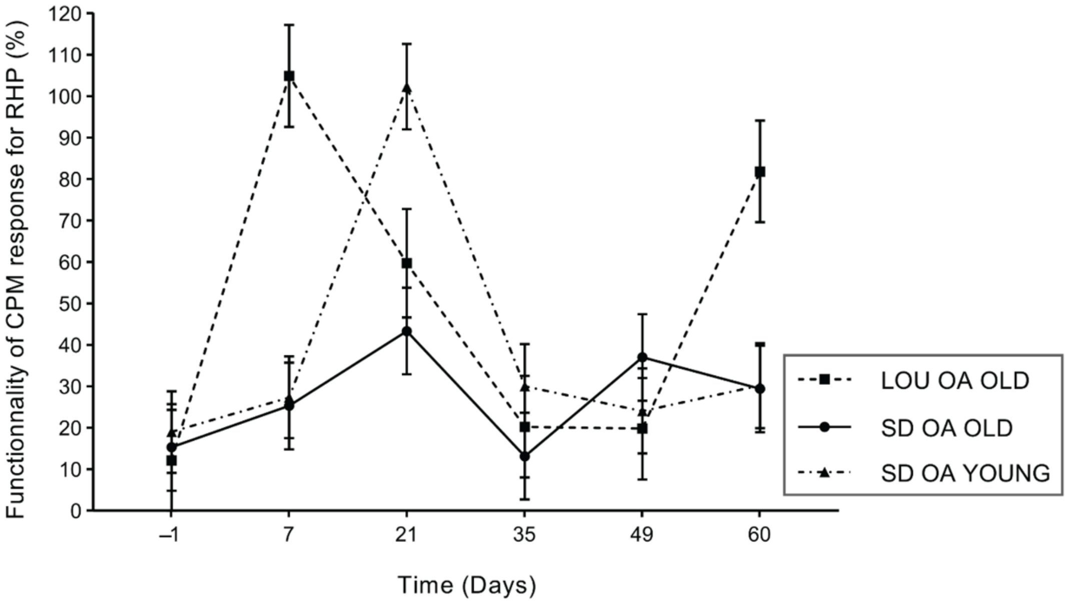

Figure S2. Percentage of change between tactile paw withdrawal threshold (PWT) post- and pre-conditioning stimulus (CS) for right-hind paw (RHP) by day (D) in LOU old (*n* = 8; 11–15 months) and SD old (*n* = 11; 11–15 months) OA groups in comparison with SD young (*n* = 12; 2–4 months) OA group. Descriptive data (mean (standard deviation)).
